# LUMI-PCR: an Illumina platform ligation-mediated PCR protocol for integration site cloning, provides molecular quantitation of integration sites

**DOI:** 10.1186/s13100-020-0201-4

**Published:** 2020-02-04

**Authors:** Joanna C. Dawes, Philip Webster, Barbara Iadarola, Claudia Garcia-Diaz, Marian Dore, Bruce J. Bolt, Hamlata Dewchand, Gopuraja Dharmalingam, Alex P. McLatchie, Jakub Kaczor, Juan J. Caceres, Alberto Paccanaro, Laurence Game, Simona Parrinello, Anthony G. Uren

**Affiliations:** 1 MRC London Institute of Medical Sciences (LMS), Du Cane Road, London, W12 0NN UK; 2 Institute of Clinical Sciences (ICS), Faculty of Medicine, Imperial College London, Du Cane Road, London, UK; 3 Imperial College Healthcare NHS Trust, London, UK; 4 Samantha Dickson Brain Cancer Unit, UCL Cancer Institute, WC1E 6DD, London, UK; 5 UCL Cancer Institute, WC1E 6DD, London, UK; 6 Centre for Systems and Synthetic Biology, Department of Computer Science, Royal Holloway, University of London, London, UK

**Keywords:** Genomics, Genetics, Mutagenesis, Retroviruses, Integration site cloning

## Abstract

**Background:**

Ligation-mediated PCR protocols have diverse uses including the identification of integration sites of insertional mutagens, integrating vectors and naturally occurring mobile genetic elements. For approaches that employ NGS sequencing, the relative abundance of integrations within a complex mixture is typically determined through the use of read counts or unique fragment lengths from a ligation of sheared DNA; however, these estimates may be skewed by PCR amplification biases and saturation of sequencing coverage.

**Results:**

Here we describe a modification of our previous splinkerette based ligation-mediated PCR using a novel Illumina-compatible adapter design that prevents amplification of non-target DNA and incorporates unique molecular identifiers. This design reduces the number of PCR cycles required and improves relative quantitation of integration abundance for saturating sequencing coverage. By inverting the forked adapter strands from a standard orientation, the integration-genome junction can be sequenced without affecting the sequence diversity required for cluster generation on the flow cell. Replicate libraries of murine leukemia virus-infected spleen samples yielded highly reproducible quantitation of clonal integrations as well as a deep coverage of subclonal integrations. A dilution series of DNAs bearing integrations of MuLV or piggyBac transposon shows linearity of the quantitation over a range of concentrations.

**Conclusions:**

Merging ligation and library generation steps can reduce total PCR amplification cycles without sacrificing coverage or fidelity. The protocol is robust enough for use in a 96 well format using an automated liquid handler and we include programs for use of a Beckman Biomek liquid handling workstation. We also include an informatics pipeline that maps reads, builds integration contigs and quantitates integration abundance using both fragment lengths and unique molecular identifiers. Suggestions for optimizing the protocol to other target DNA sequences are included. The reproducible distinction of clonal and subclonal integration sites from each other allows for analysis of populations of cells undergoing selection, such as those found in insertional mutagenesis screens.

## Background

Ligation-mediated PCR methods have diverse applications in identifying the integration sites of a known DNA sequence at an unknown locus. Applications include studying the integration site preferences of mobile genetic elements, the identification of transgene integration sites and the study of how remobilized endogenous genetic elements contribute to evolution and/or tumour development (reviewed in [1–3]). Furthermore, the ability of some mobile elements to retain activity between species, phyla, and even kingdoms, has led to a proliferation of their use for transgene delivery, gene trapping and mutagenesis screens. Additional file 1: Table S1 lists a sampling of studies and research tools that employ these techniques in organisms as diverse as bacteria, yeast, plants, nematodes, insects and vertebrates.

In most protocols, DNA is either restriction-digested or sheared and then ligated to adapters at both ends. The breakpoint between the integrated DNA and genome can then be amplified independently of the remainder of the genome, by using a primer specific to a known integrated DNA sequence and another specific to the adapter. In many protocols the use of non-complementary forked or bubble adapters, such as vectorette and splinkerette, limits the first round of DNA synthesis to the target sequence primer [4, 5]. Only after this has occurred can the adapter primer bind to a template and give rise to exponential amplification of target regions. Chemical blocking of a shortened lower strand adapter 3′ terminus can also be incorporated such that it is unable to act as a primer for template extension [6].

We recently completed a study cloning the retroviral integrations from lymphoid malignancies of hundreds of mice infected with murine leukemia virus (MuLV) [7]. Estimating the relative abundance of each mutation is important in these studies since these tumours consist of one or more dominant clones with clonal integration sites, alongside thousands of low clonality integrations that are either present in subclones of a major clone or in adjacent non-malignant tissue. Many studies have used the number of sheared DNA fragment ends to quantify the abundance of each integration [8–10]; however, one caveat of this approach is that it can lead to underestimation of highly clonal integrations as sequence coverage reaches saturation.

Here we describe LUMI-PCR (**L**igation-mediated **U**nique **M**olecular **I**dentifier **PCR**), a protocol which integrates Illumina dual index library construction with splinkerette based ligation-mediated PCR. The adapters are compatible with a standard Illumina dual index sequencing recipe and can be used to estimate the relative abundance of integrations through the incorporation of unique molecular identifiers (UMIs). Importantly, the adapters, primers and reagents can be ordered by the user to keep costs low, allowing economic processing of hundreds of samples. We have processed samples in a 96 well format using a standard liquid handling workstation (both the Beckman Biomek FX and Biomek DX models) at an overall reagent cost of US$20/£16 per library.

In this study we present integration site cloning of spleen DNA samples from mice infected with MuLV in addition to DNA derived from clonal cell lines infected with the piggyBac transposon. The protocol yields highly reproducible results with a sensitivity that allows cloning of over a thousand integration sites from a sample of 1μg of input DNA. Clonal outgrowths of cells within MuLV samples give rise to highly clonal integrations and their relative abundance can be quantitated with a high degree of reproducibility. By generating a series of libraries where DNA samples are diluted into each other at defined concentrations, we also see this quantitation reflects the known relative abundance of integrations in a complex mixture.

## Results

### Merging Illumina sequencing library adapters with a forked LM-PCR adapter that incorporates both indexes and unique molecular identifiers

**Fig. 1:**
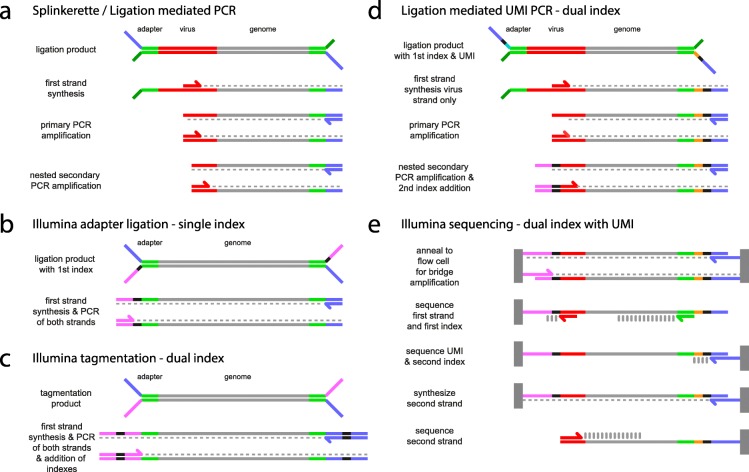
Comparison of LUMI-PCR with regular Illumina dual index library prep and with regular splinkerette PCR library prep. **a**) The steps of a traditional ligation-mediated PCR strategy using adapters with non-complementary segments and two rounds of nested PCR (e.g. splinkerette). The adapter strands are partially non-complementary and the lower strand (dark green) has no complementary primer. The adapter primer (blue) cannot bind to a template until the first strand has been synthesised from the virus primer (red). Subsequent steps will amplify virus flanked genomic regions but not other regions. **b**) Standard Illumina library preparation protocols for single index libraries. Using ligation of adapters, an index (black) is included in the adapter for each library, with one copy per fragment being present in the final product. Both strands are amplified yielding different termini at each end for flow cell binding (blue & purple). **c**) Illumina Nextera library prep using tagmentation. Adapters are added via Tn5 transposase. Both strands are amplified simultaneously using primer pairs that add an index at each end. **d**) LUMI-PCR is a hybrid protocol for ligation-mediated PCR that uses one index in the adapter and one in the secondary PCR step. A unique molecular identifier (UMI orange) is included adjacent to the adapter index (black) for quantitation of library fragments. The placement of the index is switched from the strand normally used in Illumina adapters such that it will be retained after the first strand synthesis from the virus primer. The flow cell binding sequence normally present in the Illumina adapter (purple) is included in the LTR primer of the secondary PCR amplification. **e**) A modified dual index Nextera sequencing protocol is used with custom primers and modified numbers of bases read from each index depending on the length of the custom index and the UMI (our protocol uses 10 bp indexes and an 8–10 bp UMI). The custom virus primer can be nested back from the virus genome junction to allow the junction to be sequenced

The protocol was initially developed to amplify outward from the 5′ end of the MuLV long terminal repeat (LTR) and we have also adapted it to clone the 5′ end of piggyBac transposon integrations. A custom adapter includes an 8 or 10 base pair UMI and a sequence that binds the Illumina flow cell at the initial ligation step (Fig. 1 and Additional file 1: Figure S1). In this design, unlike standard Illumina adapters, the PCR primers have no template to bind until after the first strand has been synthesized, similar to the approach used for vectorette/splinkerette PCR protocols [4, 5]. The secondary PCR primer against the LTR sequence also incorporates a sequence that is capable of binding the flow cell.

Cluster recognition on Illumina sequencers requires sequence diversity between clusters for the first 10 bp of read 1. For this reason, when compared to standard Illumina adapters, the non-complementary adapter fork sequences that hybridize each strand to the flow cell are swapped, yielding a template orientation where read 1 runs from the adapter sequence directly into the sheared end of the genomic DNA, thus guaranteeing sequence diversity between clusters. Read 2 can then be sequenced from the integration-genome junction so that all clusters can have identical bases without interfering with cluster recognition. (Fig. 1 and Additional file 1: Figure S1). The read 2 primer is offset back from the integration-genome junction to include bases spanning the junction. PCR fragments resulting from non-specific primer binding sites that do not contain an integration-genome junction can then be discarded prior to mapping. The adapter and secondary PCR integration primer both include a unique 10 bp index and the combination of these allows hundreds of samples to be pooled on a single flow cell and demultiplexed (adapter oligonucleotides are summarized in Additional file 2: Table S1).

DNA is sheared using a Covaris sonicator and libraries are purified and size-selected using magnetic bead-based purification between ligation and PCR steps. Sequencing is performed using a modified Illumina paired-end dual index recipe. The first index read is lengthened to 18 or 20 bp so that it includes 10 bp of the adapter index and 8–10 bp of the UMI. A 10 bp read is used for index 2.

After demultiplexing, read pairs are filtered by average Q-value and the beginning of read 2 is used to verify the presence of an expected integration-genome junction. Read pairs passing these criteria are retained. The bases on the integration side of the junction are trimmed and the adapter and primer sequences are trimmed. Trimmed reads are then mapped using Magic-BLAST [11] against both the genome and the sequence of the virus/transposon. Mapped read pairs are retained if they have the expected orientation within 1000 bases of each other. Pairs that map more accurately to the integration sequence than the genome, such as those resulting from internal LTR amplification, are eliminated from further analysis. Integration contigs are then built by grouping mapped reads using hierarchical clustering of the read 2 integration-genome junction coordinates.

### Hundreds of integration sites can be cloned from a single library per DNA sample

DNA was extracted from the enlarged spleen of a mouse infected with MuLV and this was processed as four replicate libraries in a 96 well format using a Beckman Biomek station on four separate occasions. Libraries were sequenced on an Illumina HiSeq 2500, and reads analysed using the pipeline summarized in Additional file 1: Figure S2. Each of the ligations yielded between 67,000 and 159,000 read pairs that passed filtering and were properly mapped to the genome. By comparison control DNAs of uninfected mouse and human samples that were processed on the same 96 well plates yielded between 0 and 13 read pairs that mapped to the genome. After contig building the resulting integration numbers ranged from 317 to 1186 per library (Additional file 1: Table S2).

The number of unique sheared-DNA fragment lengths for each integration is estimated from the number of unique mapping boundaries at the beginning of read 1 at the far end from the integration-genome junction. The number of DNA fragments per integration is also estimated using the unique number of UMI sequences for each integration. Potential sources of error exist for quantitation using either approach. Depending on coverage, the number of possible fragment lengths present for each integration is less than the number of possible UMIs per sample when using an 8 bp or 10 bp UMI. Fragment length counts may also be skewed by PCR errors, sequencing errors, end repair bias and read trimming/mapping errors that alter the mapping boundaries. Similarly, UMI numbers may be overestimated due to PCR errors (such as hybridization of unligated adapter to PCR products) and sequencing errors that introduce additional variation between UMIs. To counter this, UMIs are grouped using a Hamming distance of 1 i.e., if two UMIs differ by a single base they are counted as a single DNA fragment. This reduces 4^8^ potential combinations to 4^7^ (i.e., 16,384) or 4^10^ potential combinations to 4^9^ (262,144).

**Fig. 2:**
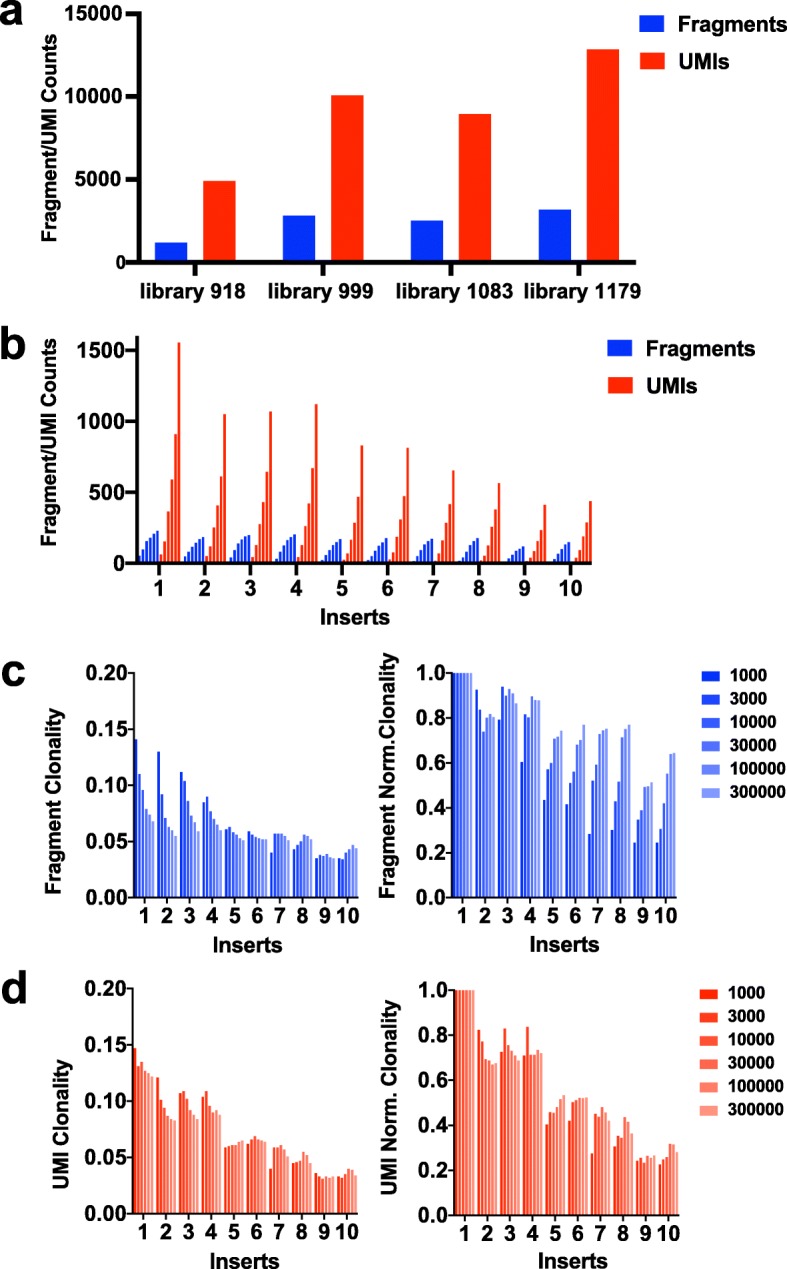
Quantitation of integration abundance and number is a function of sequencing coverage. **a**) The total number of sheared fragment length counts (blue) is substantially lower than the number of UMI counts (red) in each of four replicate libraries. **b**) A single library (#1179) was reanalysed using subsets of read pairs (1000, 3000, 10,000, 100,000 and 300,000 read pairs). Quantitation of the ten most clonal integrations for each of these subsets is shown using unique sheared fragment lengths identified per integration (blue) and UMI counts per integration (red). These values are similar when sampling lower numbers of reads but as the sample size increases, the sheared fragment length counts becomes saturated. **c** & **d**) The clonality and normalized clonality calculations for the ten most clonal integrations is calculated for all read subsets using fragment length counts (c) and UMI counts (d). For the lowest samplings (1000 & 3000 read pairs) the clonality and normalized clonality based on fragments (Fig. 2c) and UMIs (Fig. 2d) are very similar whereas a larger number of reads leads to underestimation of fragment length clonality for the most abundant inserts and conversely an overestimation of fragment normalized clonality for less abundant inserts

Fig. 2a displays the total number of sheared fragments and UMIs identified per sample. Saturating coverage of clonal integrations with hundreds of thousands of reads leads to lower estimates of sheared fragment number relative to UMI number. To dissect how this discrepancy is a function of coverage we reanalysed a single library (# 1179) using a series of subsamples of the total set of read pairs i.e. 1000, 3000, 10,000, 100,000 and 300,000 read pairs. Fig. 2b shows the fragment and UMI counts of the 10 most abundant integrations over the series of read subsamples. For the 10 most clonal integrations within this library, analysing the fewest read numbers (1000 or 3000) yields near identical numbers of sheared-fragment lengths and UMIs, but as sequencing saturation increases, UMI counts continue to increase whereas fragment length counts reach saturation.

This difference effects estimates of relative abundance of integrations as expressed as clonality i.e. the number of fragment lengths/UMIs for a single integration divided by the total number of fragment lengths/UMIs identified per sample. Normalized clonality is calculated so that the most-abundant integration has a value of 1 i.e. all clonality values are divided by the highest clonality value in the sample. For samplings with only 1000 or 3000 read pairs, the clonality and normalized clonality based on fragments (Fig. 2c) and UMIs (Fig. 2d) are very similar. Larger number of reads leads to underestimation of sheared-fragment length clonality and an overestimation of sheared-fragment length normalized clonality. A notable finding of the above analysis is that any skewing produced in sheared-fragment end counts due to oversaturation of sequencing could potentially be addressed by subsampling the total number of reads, although this effectively reduces the number of low clonality integrations identified and increases the sampling error of quantitation. The informatics pipeline provided calculates fragment numbers, UMI numbers (with and without hamming distance) and read numbers for every integration.

### Quantitation of clonal integrations is highly reproducible between libraries

**Fig. 3:**
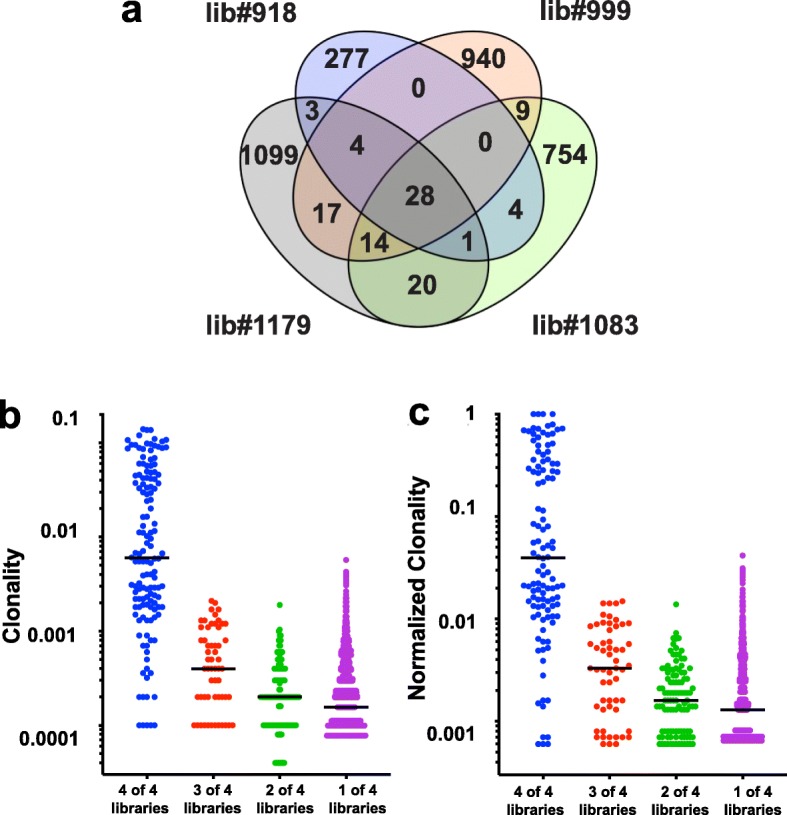
The most-clonal integrations are found reproducibly in all replicate libraries. **a**) A four-way Venn diagram illustrates the number of integrations that are found reproducibly in 1, 2, 3 and 4 replicate libraries. The majority of single fragment/subclonal integrations are only found in one library whereas the most-clonal integrations are found in all four libraries. The clonality values **b**) and normalized clonality values **c**) of all integrations were compared for integrations that were found in 1, 2, 3 and 4 replicate libraries. The set of mutations present in only one of the four libraries had substantially lower median clonality/normalized clonality values than those inserts found in more than one library. Although the vast majority of subclonal mutations were found in only one library, a fraction are also found in more than one library. All integrations with clonality > 0.01 and normalized clonality > 0.1 were found present in all four libraries

MuLV infected tissues are a complex mix of integrations derived from clonal outgrowths with subclonal components, alongside non-tumour cells that also bear integrations. Fig. 3 summarizes the overlap of integrations found in each of the four replicate libraries. A small sub fraction of mostly clonal integrations is found reproducibly between libraries, with 28 integrations being found in all four libraries (Fig. 3a). When plotting the clonality and normalized clonality of integrations that are found in 1, 2, 3 and 4 of the replicate libraries there is a clear trend whereby the least reproducible integrations present in 1, 2 or 3 libraries have a lower clonality than integrations identified in 4/4 libraries. All integrations with clonality > 0.01 and normalized clonality > 0.1 are found in all four libraries (Fig. 3b & c).

**Fig. 4:**
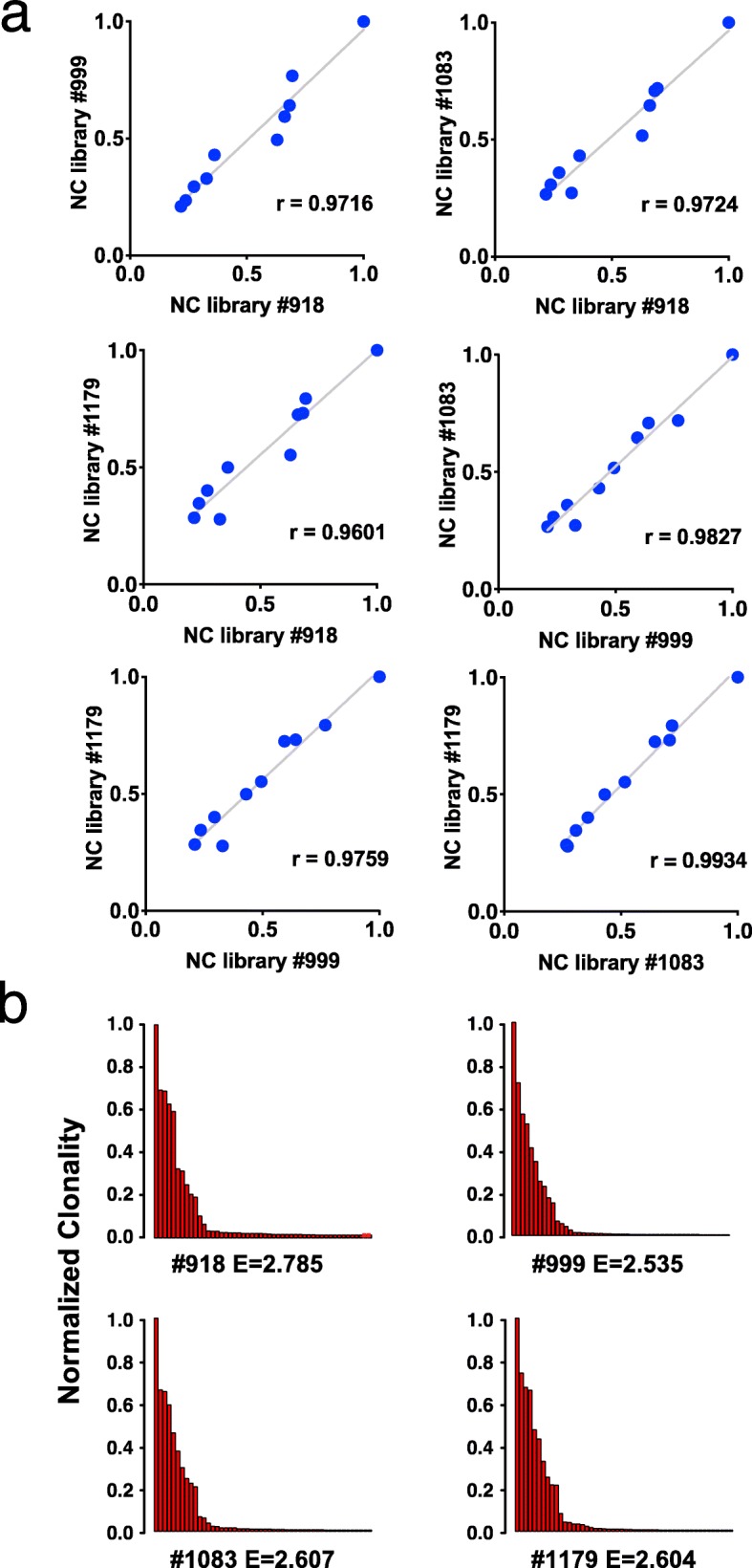
Quantitation of the 10 most-clonal integrations is highly reproducible between libraries. **a**) Spearman correlation coefficients were calculated for pairwise comparisons between all 4 replicates using normalized clonality (NC) values for the 10 most-clonal integrations. Rho values range between 0.9601 and 0.9934. **b**) Normalized clonality profiles of the top 50 most-clonal integrations from each sample are highly similar, with a narrow range of entropy values between 2.535 and 2.785

Quantifying the relative abundance of integrations with respect to one another is important for insertional mutagenesis screens in order to differentiate the most-selected integrations of clonal outgrowths from weakly selected or unselected mutations. Fig. 4a depicts pairwise scatterplots comparing normalized clonality of the 10 most-clonal integrations in each of the four replicate libraries. We observe a high degree of reproducibility as measured by Pearson correlation coefficients (rho values ranging from 0.9601 and 0.9934). The degree of clonal outgrowth observed in a polyclonal mixture of cells can be measured using the Shannon entropy [12–14]. We calculate this value for MuLV tumour samples using the normalized clonality values of the 50 most-clonal integrations, depicted in Fig. 4b. The entropy values for the four replicate libraries fall within a narrow range from 2.535 to 2.785.

### Quantitation of integrations is linear over a range of concentrations

**Fig. 5:**
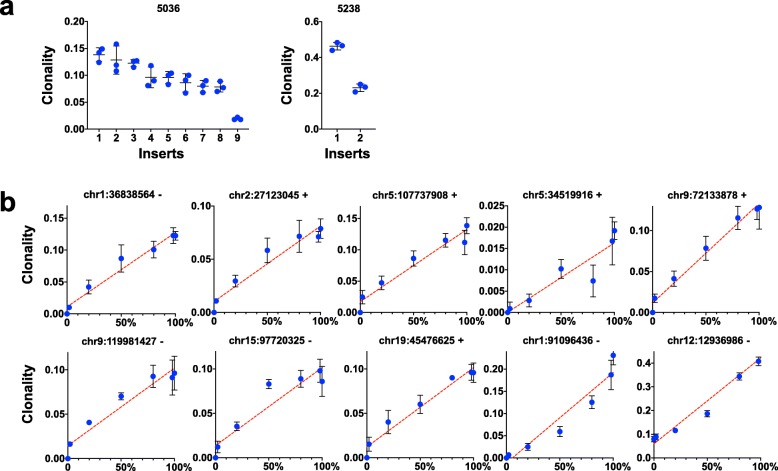
Quantitation of MuLV integrations over a range of concentrations. **a**) Triplicate libraries were analysed from two MuLV infected spleen DNA samples, identifying nine clonal integrations in sample #5036 and two clonal integrations in sample #5238. Integration 9 from sample #5036 and integration 1 from sample #5238 both map to the same base pair in the 3′ UTR of *Mycn* (chr12:12936986) which is a highly selected hotspot for integrations in MuLV infected lymphoma samples. Triplicate libraries of uninfected DNA did not contain any mappable reads. **b**) These two DNAs were mixed with each other at ratios of 1:49, 1:4, 1:1, 4:1 and 49:1 and triplicate libraries were constructed. The clonality of each of the integrations is plotted against the percentage of its source DNA present in each mixture. Plots 1–8 are inserts 1–8 from sample #5036. Plot 9 is insert 2 from sample #5238. Plot 10 simultaneously represents insert 9 from sample #5036 and insert 1 from sample #5238

Because the relative abundance of integrations can span orders of magnitude, it is important to verify that quantitation reflects the known starting concentration of integrations within a complex mixture. To this end we prepared two dilution series of mixed DNAs with replicate libraries prepared from each series. The first series uses DNAs extracted from two MuLV infected spleens. Triplicate libraries were constructed from the individual DNAs, a series of mixed DNAs and controls of uninfected DNA. The uninfected DNA controls yielded no reads that mapped to the mouse genome. Libraries from individual DNAs yielded highly reproducible quantitation of 9 and 2 clonal integration sites each, in addition to many low abundance or single read integration sites (Fig. 5a). A series of reciprocal dilutions of 2-fold, 5-fold and 50-fold demonstrated a linear concentration dependent correlation between dilution factor and integration quantitation (Fig. 5b). One of these integrations at the highly recurrent *Mycn* 3′ UTR locus was present in both samples at different clonality and the linearity of dilution series is also preserved for this integration.

**Fig. 6:**
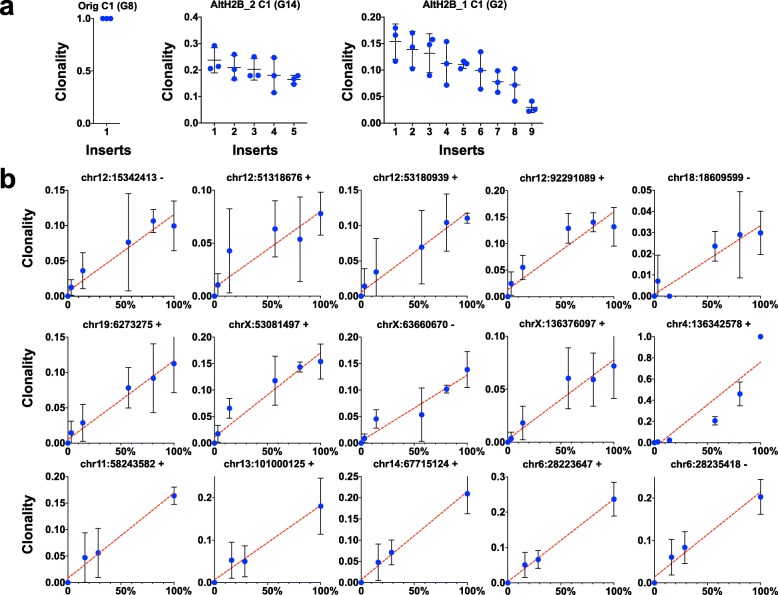
Quantitation of piggyBac integrations over a range of concentrations. **a**) Triplicate libraries were analysed from three cell lines derived from mouse neuronal precursors transfected with piggyBac and cloned by single cell sorting. These DNAs have 1, 5 and 9 integrations each. Triplicate libraries of uninfected DNA did not contain any mappable reads. **b**) These three DNAs were mixed with each other at ratios of 1:2:4, 4:2:1, 1:5:25 and 25:5:1 and triplicate libraries were constructed. The clonality of each of the integrations is plotted against the percentage of its source DNA present in each mixture. Plots 1–9 are inserts from cell line AltH2B_1 C1 (G2). Plot 10 is the insert from cell line Orig C1 (G8). Plots 11–15 are the inserts of the sample AltH2B_2 C1 (G14)

For the second dilution series, mouse neuronal precursors were infected with a piggyBac transposon vector and these were single cell sorted to grow clonal cell lines. Triplicate libraries were constructed using three cell lines alongside non-transfected control mouse DNA. PCR primer binding sites were chosen against the 5′ ITR taken from the previously published QIseq protocol [15]. The three cell lines contained one, five and nine integrations each (Fig. 6a) whereas untransfected DNA gave no reads mapping to the mouse genome. Even with clonal cell lines there is some variance in the abundance of integrations; in the third cell line one of the nine integrations is poorly amplified. This likely reflects site and sequence specific influence of shearing and/or PCR amplification. Four mixtures of DNAs were created to give a range of concentrations of all three DNAs and triplicate libraries were constructed from these mixtures (Fig. 6b). Quantitation is consistently linear in a concentration dependent manner over ranges of 3% up to 100%.

## Discussion

The integration site cloning methodology presented here uses a novel Illumina/ligation mediated PCR hybrid adapter that includes UMIs and limits the total amplification cycles to two nested PCRs of 16 cycles (reduced from 50 total cycles in our previously published Splinkerette protocol [16]). The informatics pipeline allows quantitation by both UMIs and sheared fragment lengths. Although increased sequencing coverage per library yields a higher number of single fragment low clonality integrations, there is a trade off in that saturating sequencing coverage can skew quantitation by fragment length. As sequence coverage approaches saturation, the use of UMIs increases the likelihood that final quantitation is more representative of the initial sample at the ligation stage. Furthermore, we demonstrate that saturation of sheared-fragment length quantitation of clonal reads can be mitigated by subsampling the reads used for analysis. Quantitation of integrations is highly reproducible and linear over a series of dilutions where the starting concentration of each integration is known.

Although the protocol was extensively optimised for cloning MuLV integration sites, we have shown it can also be applied to other integrants such as the piggyBac transposon. When optimizing new conditions, we have found it is useful to test and compare a series of PCR primers, cycle numbers and annealing temperatures as well as DNA extraction methods. The number of PCR cycles should be minimised to reduce the effects of amplification bias [17] however the cycle number used must meet a theoretical minimum based on the relative abundance of the target sequences relative to the entire genome size. For 1 μg of genomic DNA with a single clonal integration per mammalian cell the theoretical minimum number of amplification cycles to produce 1 ng of library (2.32 × 10^9^ copies) is 14 cycles but in practice we use 32. We have generally found that higher cycle numbers improve the fraction of cluster forming, mappable PCR products, although this potentially increases amplification bias. More cycles would be justified where accurate quantitation of clonal outgrowth is less of a priority than maximal sensitivity amplifying an unselected population of cells containing primarily subclonal inserts. PCR conditions can be compared by qPCR using SYBRgreen and multiple PCRs prepared with different cycle numbers can then be pooled and sequenced on a MiSeq to examine which conditions yield the highest number of mappable sequences compared to unmappable/unsequencable PCR artefacts.

The MuLV long terminal repeat (LTR) sequence is very similar to hundreds of endogenous retrovirus like sequences, therefore our primers were carefully chosen for their lack of sequence similarity to endogenous sequences at the primer 3′ end and their inability to amplify products from uninfected control DNAs. Two rounds of nested PCR are followed by sequencing using a further nested primer, and any mismatch of endogenous sequences with these nested primers helps reduce the background of endogenous sequence PCR products and/or prevents these products from yielding high quality sequence. It is possible that other DNAs with no similarity to endogenous sequences may require less nesting of PCR or sequencing primers.

The binding site of primers should be near enough to the integration-genome junction to maximise the genomic sequence that can be mapped but far enough from the junction for nesting of the PCR and sequencing primers. The sequencing primer should ideally leave enough bases to give unambiguous identification of the integration-genome junction. We have found a 10 bp offset from the end of an MuLV integration is more than sufficient to give an unambiguous integration-genome junction sequence.

The LTR sequences present in many retroviruses, retrotransposons and viral vectors are tandem duplicated at either end of the integrant, meaning 50% of all amplified fragments will be internal to the virus/vector and not give information about the integration site. These fragments can be removed by using a restriction enzyme site close to the end of the internal LTR repeat to cleave all ligation fragments that might be amplifiable from the internal primer binding site. Other researchers have employed the use of a locked nucleic acid primer that binds and blocks amplification of the internal fragments [18]. An alternative strategy is to simply ignore these unwanted internal sequences and allow sequencing of the internal sequences to use 50% of all reads. This last approach was used for the data in Figs. 5 and 6. The informatics pipeline maps reads to both the virus/transposon sequence and the genome. Reads mapping better to the virus/transposon are then excluded.

Contamination between samples is a relatively minor concern for standard sequencing libraries where amplification is a single final step, and where all target products are of similar abundance. When performing ligation mediated PCRs however, less than 0.01% of the genome is targeted for amplification and when comparing subclonal and clonal integrations, the abundance of target products spans orders of magnitude. Aside from contamination between the initial DNA samples and libraries, the greater concern is contamination of pre-amplification material and reagents with post-amplification products, which will readily dominate all subsequent PCR reactions. As such control DNAs are essential and should ideally yield no reads that map to the genome of interest.

To minimise contamination between starting DNA samples, tissues are dissected with instruments that are cleaned between uses by soaking in DNA-ExitusPlus and then autoclaved. DNA is extracted, and libraries are ligated and size selected, in pre-PCR conditions. The first round of PCR is carried out on one thermal cycler, the primary PCR is cleaned in a second lab (we use a second set of pipettes and or liquid handling station), and the second PCR is carried out on another thermal cycler in a third lab. Filter tips are used for all steps of library preparation. The primary and secondary PCR products are never handled in the same room as the starting material or as each other. The 96 well head of the Biomek liquid handling workstations can be disassembled and treated with DNA-ExitusPlus between runs. UV lamps can also be employed to degrade stray PCR products.

For liquid handling of large numbers of samples in 96 well plates, efforts should be made to prevent aerosol contamination between samples. Where only moderate numbers of samples are being processed (24 to 48 in a plate) alternating rows and/or columns should be left empty. Removal of strip caps or unpeeling of adherent lids increases the possibility of aerosol creation, therefore the use of foil plate lids that can be pierced by a pipette tip is strongly recommended. Transfer of samples with a single channel pipette may be preferable to the use of a multichannel pipette to prevent tips from binding to pierced adhesive lids and coming loose from the pipette. Pre-piercing lids with one tip and pipetting with a second tip may also be helpful.

We have included protocols (.xps files, Additional file 3) and plate layouts for the Beckman Biomek liquid handling workstations. These customised programs were designed to minimise creation of drips and aerosols through the use of slow pipetting head movement, frequent tip touches to the side of the wells and gradual pipetting at or just below the liquid surface. This is particularly important when using suspensions of magnetic beads in polyethylene glycol, which when mixed with ethanol can create bubbles at the ends of tips that need to be removed by tip touching on the side of the 96 well plate.

For elements that have unavoidable similarity to endogenous sequences (such as studying remobilization of endogenous elements) amplification of unwanted sequences may be unavoidable and these will need to be identified and discarded at the informatics stage. Similarly, recurrent PCR artefacts and/or cross contamination between samples should be identified and discarded. Our pipeline identifies integrations that are found at the same position in the same orientation between samples and differentiates between duplicate integrations that are expected (in replicate samples) and duplicates that are not expected between samples of a different origin or in uninfected controls.

When analysing tumours, it is important to recognise that infected cell clones may circulate or metastasize between organs. As such, finding the same integration profile in multiple samples from the same mouse does not necessarily mean contamination has occurred. Therefore, if filtering for recurrent integrations found in multiple PCRs, the integrations for all samples originating from the same mouse should be pooled before comparison between samples. Furthermore, some viruses/transposons/mobile elements have highly recurrent integration sites (e.g. in MuLV tumours the 3′ UTR of the *Mycn* locus).

Numerous approaches are used to identify regions of the genome where selection for integrations has taken place. The CIMPL/KCRBM pipeline uses kernel-based density estimates of integration distributions compared to random permutations [19, 20]. TAPDANCE uses Poisson distribution statistics to estimate significant selection, taking into account the distribution of integrations to the distribution of possible integration sites (TA or TTAA motifs for piggyBac and Sleeping Beauty respectively) [21]. Strand bias of integration sites (in the forward or reverse orientation) can indicate selection for integrations that activate/inactivate genes at a specific locus [22, 23]. For remobilization of endogenous elements, the integration profiles of multiple independent samples are compared for evidence of sample specific de novo integrations [24].

We have previously compared the distribution of integrations in samples undergoing selection/clonal outgrowth with integration profiles of samples collected shortly post infection [7]. This and other studies indicate that entropy is a useful measure of the degree of selection that has taken place within a DNA sample. The final script in our pipeline includes entropy calculations for each sample.

The question of how to separate integration biases from changes in integration abundance due to selection could potentially be addressed at the molecular level. Other studies have used a unique “serial number” sequence within the genome of the mobile element itself. In experiments using the Tf1 retrotransposon in *S. pombe*, an 8 bp random sequence was introduced into the U5 region of the 5′ LTR [25, 26]. A complex library of transposons with serial numbers is then introduced to cells, effectively assigning a unique identity to each element at the time of integration. Quantitation of integration biases can thus be observed independently of selection of these events after the time of integration. This approach is best suited to mobile elements that are introduced to target cells in large numbers simultaneously. Although the approach is less suited to MuLV integrations that are derived from virus that replicates in vivo or to experiments where the transposable element is carried in the germline prior to mutagenesis, it could be used in any system where the mobile element is introduced as a sufficiently complex library. Integration site biases could then be analysed by unique serial number for each integration and selection of each unique integration could be independently quantitated by adapter UMIs and/or ligation fragment lengths.

## Conclusion

We present here an integration cloning protocol that minimises PCR cycle number. Incorporating UMIs into the initial adapter allows quantitation that is less prone to saturating sequence coverage than the use of sheared fragment lengths. The protocol additionally maintains the diversity of complex mixtures of low abundance integrations. Although optimized for MuLV LTR sequences, we have also applied it to the use of piggyBac transposons and it could potentially be applied to other mobile genetic elements such as those listed in Additional file 1: Table S1. Furthermore we demonstrate here and in our prior study [7] the practicality of scaling sample numbers into the hundreds through the use of an automated liquid handling workstation.

## Method

### DNA extraction

For mammalian tissues and cells DNA was extracted with Qiagen Allprep and Qiagen Puregene kits. Tissue fragments are homogenized using a motorized handheld grinder (Sigma #Z359971-1EA) with disposable polypropylene pestles (Sigma #Z359947) with a pestle tip that fits standard microfuge tubes. DNA was diluted to 20 ng/μl in PCR grade water. DNA quantitation of input material and final library normalization was performed using fluorescent dsDNA dyes with a standard curve of control DNA samples e.g. picoGreen quantitation using a fluorometer plate reader or a Qubit fluorometer.

### DNA shearing, blunting and A-tailing

Transfer 58 μl of each diluted DNA sample at 20 ng/μl (total ~ 1.1 μg DNA) into a Covaris 96 microTUBE plate (520078) and cover with a foil seal (transfer takes ~ 45 min). Load the microTUBE plate containing DNA onto the sonicator. Fill the water bath of the Covaris E220 Sonicator (with E220 Intensifier included) and de-gas for at least 45 min prior to shearing. Shear all wells containing DNA using the settings: Peak Incident Power 175 watts, Duty Factor 10%, Cycles per Burst 200, Treatment Time 180 s, Temperature range 0–40 °C. After shearing, the DNA should typically have an average length of 400 bp, which can be confirmed using the Agilent Bioanalyser HS DNA assay or by running on a 2% agarose gel with ethidium bromide.

DNA is blunted to create 5′-phosphorylated blunt ends. Defrost and touch centrifuge the plate of sheared DNA, pierce foil of each well and transfer 52.5 μl of sheared DNA into a new conical 96 well plate (Cat#30128575). Prepare a master mix of NEBNext® End Repair Module (NEB; E6050L) and divide evenly into 8 or 12 wells of a PCR tube strip. Using a multichannel pipette add 24.5 μl of master mix to each well containing sheared DNA.

**Table:** 

	μl per sample	μl for 96 well master mix (× 110)
DNA	52.5	*
10x reaction buffer	7.7	847
End Repair Enzyme Mix	4	440
H_2_O	12.8	1408
Total	77	2695

Cover plate with a foil seal, vortex and touch centrifuge. Incubate on thermal cycler in pre-PCR room at 20 °C for 30 min (no inactivation step is required). Touch centrifuge the plate, pierce the foil of each well and transfer 77 μl of blunted DNA into a new conical 96 well plate.

Use magnetic beads and ethanol to clean the DNA (this step is repeated below several times during library preparation). Prepare 100 ml of fresh 80% ethanol. Vortex Agencourt AMPure XP magnetic beads thoroughly (Beckman Coulter; A63880) immediately prior to use. Add 90 μl of beads to each well and mix the DNA and beads slowly by pipetting up and down. Incubate plate for 10 min. Place plate on 96 well magnet for 10 min. Remove and discard the supernatant. Remove the plate from the magnet. Add 100 μl of fresh 80% ethanol and mix slowly by pipetting up and down. Place plate on 96 well magnet and incubate for 10 min. Remove and discard supernatant and remove the plate from magnet. Repeat the ethanol wash step and remove and discard the supernatant. Allow beads to dry for 5 min. Add 50 μl of distilled water, incubate for 2+ minutes and place plate on magnet for 10 min. Collect 42 μl of supernatant into a clean PCR plate, being careful to avoid disturbing the magnetic bead pellet.

Adenosine nucleotide extensions are added to the 3′ ends of blunted DNA to create an A overhang for annealing the T overhang of the adapter, thus aiding adapter ligation. Touch centrifuge the plate. Prepare a master mix of NEBNext® dA-Tailing Module (NEB; E6053L) with Klenow fragment. Divide mix into 8 or 12 wells of a PCR tube strip and using a multi-channel pipette add 8 μl to each well of DNA.

**Table:** 

	μl per sample	μl for 96 well master mix (× 110)
End Repairs, Blunt DNA	42.0	*
NEBNext dA-Tailing Reaction Buffer	5.0	550
Klenow Fragments (3′ > 5′ exo)	3.0	330
Total	50.0	880

Cover plate with a foil seal, vortex and touch centrifuge. Incubate on thermal cycler in pre-PCR room at 37 °C for 30 min (no inactivation step is required). Touch centrifuge the plate, pierce the foil seal for each well and transfer 50 μl of A-tailed DNA into a new 96 well conical plate. Be careful to pipette the DNA into the bottom of the wells to avoid bubbles in subsequent pipetting steps. Repeat the EtOH washing protocol described above, eluting with 50 μl of distilled water and collecting 36 μl of supernatant containing the DNA. The cleaned A-tailed DNA plate is covered with a foil seal and can be stored at − 20 °C for later use.

### Adaptor preparation and ligation

A unique, indexed upper adaptor containing a UMI is mixed with a universal lower adapter and annealed to make non-complementary forked adapters (see Additional file 2: Table S3 for oligonucleotide sequences). All index sequences were adapted from the unique index sequences designed for maximal diversity in Xu et al. [27].

Resuspend the adapter oligonucleotides at 100 μM in H_2_O and shake intermittently over 30 min. Create diluted 10 μM stocks of each oligonucleotide (for the 96 unique upper adapters this can be done in a 96 well plate). Add a unique upper strand oligonucleotide to each well of a 96 well PCR plate. Make a master mix of the universal lower strand adapter, NEB buffer and water and add 32 μl of master mix to each well of the 96 well PCR plate. The volumes below will yield 40 μl of each adapter which is enough for 4 plates using 8 μl per ligation (accounting for evaporation and pipetting volume error).

**Table:** 

	μl per sample	μl for 96 well master mix (× 110)
Upper Strand Adaptor, 10 pmoles/μl (40 pmoles)	8	–
Universal Lower Adaptor, 10 pmoles/μl (40 pmoles)	8	880
NEB buffer 2.1	4	440
H_2_O	20	2200
Total	40	3520

Seal with a foil lid and anneal on a thermal cycler, using a 95 °C 3-min denaturing step after which the temperature is decreased by 1 °C per 15 s (4 °C per min) to 20 °C. The annealed adapters are aliquoted into a series of PCR plates adding 8 μl per well. The final adapter concentration is 4 μM. 1 μg of DNA sheared to a length of ~ 400 bp is equivalent to 3.8 picomoles per ligation (slightly less after blunting and A-tailing). 8 μl of adapter is used per ligation i.e. 24 picomoles. Therefore, the approximate molar ratio of adapter to DNA is > 6:1. It is important to prevent cross-contamination of oligonucleotide and adapter stocks. These can be stored in individual screw capped tubes kept in a 96 well rack (e.g. Micronic screw cap tubes MP52706).

Add 36 μl of A-tailed and cleaned DNA to a plate of 8 μl aliquots of adapters prepared in step 2. Make a master mix of T4 DNA Ligase (NEB; M0202 L) and ligase buffer, divide into a PCR tube strip. Using a multi-channel pipette add 7 μl to each well of A-tailed-cleaned DNA. Cover with foil seal, vortex and centrifuge. Incubate on thermal cycler in pre-PCR room 20 °C for 2 h followed by inactivation of 65 °C for 10 min.

**Table:** 

	μl per sample	μl for 96 well master mix (× 110)
DNA (~ 3.8pmoles)	36	*
Buffer	5	550
T4 Ligase (400,000 units/ml)	2	220
Unique adaptor (~40pmoles)	8	*
Total	51	770

### Restriction digestion of ligations

This step is an optional requirement for some integrations prior to amplification. It is used for MuLV or any vector/element that includes tandem repeats of LTRs at either end. Digestion is used to eliminate the fragments generated by the 5′ end of the 3′ LTR which is identical to the 5′ end of the 5′ LTR. Removing these sequences means sequencing coverage is not used on fragments internal to the vector/virus. The choice of enzyme is dependent on the sequence of the integrated DNA but the restriction site should be closer to the internal PCR primer binding site than the average fragment length of the library. A digestion step may also be useful to eliminate concatemers of transgenes produced by pronuclear injection, or concatemers of DNA transposons that have not undergone mobilization (although this requires that the concatemer has a restriction site external to the ends of inverted direct repeat). An alternate approach to this digestion step is to employ an locked nucleic acid oligonucleotide that is complementary to the unwanted internal fragment in the PCR [18]. If a digestion step is not required, the previous ligation volume should be adjusted to 60 μl for subsequent cleaning steps.

Touch centrifuge the ligation plate. Pierce the foil seal of each well and transfer 51 μl of adaptor ligated DNA into a new 96 well conical plate. Prepare a master mix of EcoRV-HF® (NEB; R3195L), CutSmart Buffer (cat#B7204S) and H_2_O. Divide the master mix into 8 or 12 wells of a PCR tube strip. Using a multi-channel pipette add 9 μl of master mix to each well of the ligation plate.

**Table:** 

	μl per sample	μl for 96 well master mix (× 110)
DNA	51	*
CutSmart Buffer	6	660
EcoRV-HF	1	110
H_2_O	2	220
Total	60	990

Cover plate with foil seal, vortex and touch centrifuge. Incubate on thermal cycler or in an incubator in the pre-PCR room at 37 °C overnight. The following day inactivate on a thermal cycler at 65 °C for 20 min.

### Size selection of ligation/digestion

Touch centrifuge the plate, pierce the foil seal of each well and transfer 60 μl of ligated-digested DNA into a new 96 well conical plate. Be careful to pipette the DNA into the bottom of the wells to avoid bubbles in subsequent pipetting steps. Add 40 μl of distilled water per well. If a digestion step was not included, add 50 μl.

Prepare 100 ml of fresh 80% ethanol. Vortex AMPure XP magnetic beads immediately prior to use. Add 60 μl of magnetic beads to each well and mix slowly by pipetting up and down. Incubate for 10 min. Place plate on 96 well magnet for 10 min. Remove 158 μl of supernatant and transfer to another conical plate. Discard the used bead plate. Add 50 μl of magnetic beads to each well and mix slowly by pipetting up and down. Incubate for 10 min. Place plate on 96 well magnet and wait 10 min. Remove and discard supernatant.

Add 100 μl of fresh 80% ethanol and mix slowly by pipetting up and down. Place plate on magnet and wait 10 min. Remove and discard supernatant and remove the plate from magnet. Repeat the ethanol wash step and remove and discard supernatant. Allow beads to dry for 5 min. Add 50 μl of distilled water, incubate for 2+ minutes and place plate on 96 well magnet for 10 min. Collect 32 μl of supernatant into a clean PCR plate, being careful to avoid disturbing the magnetic bead pellet. The size selected ligation plate can be covered with a foil seal and stored at − 20 °C for later use.

### Primary (q)PCR

Touch centrifuge the plate. Pierce the foil seal of each well and transfer 28.5 μl of digested size-selected ligation into a new 96 well PCR plate (an optical plate for qPCR). Prepare the primary qPCR master mix, divide evenly into 8 or 12 wells of a PCR tube strip. Using a multi-channel pipette add 21.5 μl to each well of the library PCR plate.

Primary PCR primers.

LTR primary PCR primer.

5′-GCGTTACTTAAGCTAGCTTGCCAAACCTAC-3′.

Adapter PCR primer.

5′-AATGATACGGCGACCACCGAGATCTACAC-3′.

**Table:** 

	μl per sample	μl for 96 well master mix (× 110)
DNA	28.5	*
HF Buffer (5x)	10	1100
10 mM dNTPs	1	110
LTR primary PCR primer (10 μM)	2.5	275
Adapter PCR primer (10 μM)	2.5	275
Phusion Hot Start II (F549S)	0.5	55
SYBR® Green I (0.1x)	5	550
Total	50	2365

Cover with either a foil or optical plate seal, vortex and touch centrifuge. Incubate on a thermal cycler in the pre-PCR room using the 1°PCR program after which the plate can be stored at − 20 °C for later use.

**Table:** 

Cycle#	Denaturation	Annealing	Extension
1	98°C for 30 sec	-	-
2-17	98°C for 10 sec	66°C for 30 sec	72°C for 30 sec
18	-	-	72°C for 5 min

If using an optical seal, prior to transfer, pierce a cross pattern into each well with a razor blade (a new blade for each well). Transfer 50 μl of 1°PCR product into a new conical plate. Be careful to pipette the DNA into the bottom of the wells to avoid bubbles in subsequent pipetting steps. Prepare 100 ml of fresh 80% ethanol and perform a magnetic bead/ethanol wash as described above. The cleaned PCR plate can be covered with a foil seal and stored at − 20 °C for later use.

Optional: Quantify the cleaned primary PCR product using picoGreen or Qubit HS dsDNA kit. The expected average concentration of PCR reactions should be 2.5 ng/μl. Where products have been quantitated approximately 50 ng (~ 20 μl) is used as template for the secondary PCR. Alternatively, 28.5 μl of all PCR products can be used as template for the next step.

### Secondary (q)PCR and clean up

The second index is added to the LTR end of the PCR products during the secondary PCR step. We use 12 different indexed 2° PCR primers per plate arranged so that no indexed primer is adjacent to itself in any direction. This ensures that any unexpected index combinations arising from cross contamination can be eliminated at the demultiplexing stage. Below is a sample layout for two rows which can be repeated for the entire plate. A new set of 12 secondary index primers is used for each plate so that multiple plates of libraries can be pooled for sequencing. Pooling up to 7 plates on a single HiSeq flow cell offers sufficient coverage for tens of thousands of reads per sample. Miseq Nano flowcells are sufficient when sequencing dozens of samples.

**Table:** 

	1	2	3	4	5	6	7	8	9	10	11	12
A	LTR 2° #1	LTR 2° #2	LTR 2° #3	LTR 2° #4	LTR 2° #5	LTR 2° #6	**LTR 2°** **#7**	**LTR 2°** **#8**	**LTR 2°** **#9**	**LTR 2°** **#10**	**LTR 2°** **#11**	**LTR 2°** **#12**
B	**LTR 2°** **#7**	**LTR 2°** **#8**	**LTR 2°** **#9**	**LTR 2°** **#10**	**LTR 2°** **#11**	**LTR 2°** **#12**	LTR 2 ° #1	LTR 2° #2	LTR 2° #3	LTR 2° #4	LTR 2° #5	LTR 2° #6

Add either 28.5 μl or 50 ng of the primary PCR DNA into 2° PCR plate and if needed adjust volume of each well to 28.5 μl with H_2_O. Add 2.5 μl of 12 unique indexed primers to each well using the pattern above. Prepare the PCR master mix and divide evenly into 8 or 12 wells of a PCR tube strip. Using a multi-channel pipette add 19 μl to each well.

LTR secondary nested PCR primer (variable index bases are indicated in bold).

5′-CAAGCAGAAGACGGCATACGAGAT**TCTGTATTTC**GCTAGCTTGCCAAACCTACAGGTGG-3′.

Primary/secondary PCR adapter end primer.

5′-AATGATACGGCGACCACCGAGATCTACAC-3′.

**Table:** 

	μl per sample	μl for 96 well master mix (× 110)
DNA (50 ng)	variable	*
H_2_O	variable	*
HF Buffer (5x)	10	1100
10 mM dNTPs	1	110
Adapter primer (10 μM)	2.5	275
LTR secondary indexed primer	2.5	*
Phusion Hot Start II	0.5	55
SYBR®Green I (0.1x)	5	550
Total	50	2090

Cover with either a foil or optical plate seal, vortex and touch centrifuge. Incubate on a thermal cycler in the pre-PCR room using the 1°PCR program.

**Table:** 

Cycle#	Denaturation	Annealing	Extension
1	98°C for 30 sec	-	-
2-17	98°C for 10 sec	66°C for 30 sec	72°C for 30 sec
18	-	-	72°C for 5 min

Transfer 50 μl of 2° PCR product into a new conical plate. Be careful to pipette the DNA into the bottom of the wells to avoid bubbles in subsequent pipetting steps. Prepare 100 ml of fresh 80% ethanol and perform a magnetic bead/ethanol wash as described above.

### Final library compilation

Quantitate the secondary PCR product using picoGreen or a Qubit HS dsDNA kit. The expected average concentration of PCR reactions is 7 ng/μl. After quantitating each sample, calculate the volume required to obtain 20 ng of each sample. We typically pool 20 ng of up to 96 samples into a single Eppendorf tube, after which the pooled library is requantitated by Qubit. Each pool is quantitated by KAPA Illumina SYBR Universal Lib Q. Kit (Anachem; KK4824) as per the manufacturer’s instructions with dilutions of each library at 1/100, 1/1000, 1/10,000. Confirm the fragment length distribution of each pool of 96 libraries using the HS DNA Chip on the Agilent Bioanalyser. Pool equal quantities of multiple tubes into a single tube and requantitate with a Qubit for loading onto a HiSeq or MiSeq flow cell.

### Sequencing

The library can be sequenced using a standard Illumina paired end dual index 2 × 100 bp recipe with minor alterations to the index read lengths.

- Read 1 primer – custom adapter primer sequencing the adapter-sheared DNA end junction.

5′- TTCAGACGTGTGCTCTTCCGATC − 3′.

- Index 1 primer (i7 equivalent) - LTR primer running toward the flow cell sequencing the LTR end index (10 cycles).

5′- TGTAGGTTTGGCAAGCTAGC − 3′.

- Index 2 flow cell primer (i5) - present on the flow cell, sequencing the 10 bp adapter index then 8–10 bp UMI (18–20 cycles).

- Read 2 primer - LTR primer reading the integration-genome junction.

MuLV Option 1 (set back 6 bp from LTR-genome junction, 5 nested bases from the secondary PCR primer).

5′- GCTAGCTTGCCAAACCTACAGGTGGGGTC − 3′.

MuLV Option 2 (set back up to 10 bp from LTR-genome junction, no nested bases from PCR primers).

5′- GCTAGCTTGCCAAACCTACAGGTGG − 3′.

### Detailed step by step protocol and liquid handling workstation protocols

The supplemental methods file gives a more detailed step by step summary of the above method and includes protocols for processing 96 well plates on a Beckman Biomek liquid handling workstation. Detailed step by step protocols can be obtained by loading the .xpl files for each protocol into the Beckman Biomek software. Additional file 1: Figure S3 includes plate layouts for these programs.

### Informatics

A summary of informatics steps is outlined below and in Additional file 1: Figure S2. A detailed step by step pipeline and scripts are available for download at https://github.com/anthonyuren/LUMI-PCR-pipeline/.

Demultiplexing using bcl2fastq is performed using custom parameters so that the second (i5) index is retained to extract the UMI information. The beginning of read 2 is examined for the integration-genome junction. Trimming of adapter sequences is performed using custom sequences corresponding to the adapter and primers. Trimmed reads are mapped using Magic-BLAST [11] and bam files are created using SAMtools [28].

Correctly paired and mapped reads are assigned genomic coordinates and by using the orientation of read 1 and read 2 the genome junctions and sheared fragment ends are identified. Some variance at the LTR-genome junction position may be observed due to sequencing/PCR errors, so mapped coordinates are then grouped into contigs by hierarchical clustering of all reads based on the LTR-genome junction position. UMI sequences are then extracted from the i5 index read and assigned to each contig. UMIs that mismatch by 1 base of the 8 bp UMI sequence (i.e., UMI pairs with a Hamming distance of 1) are collapsed into a single value since statistically these are most likely to arise from sequencing errors or PCR amplification errors. The number of unique UMIs associated to each LTR position is then used as the number of fragments for that integration.

The total number of UMIs present for each integration is summed for each library, and then the “clonality value” for each integration is calculated as the fraction of fragments for each integration relative to the sum of fragments for entire sample. In analyses of MuLV tumours the number of clonal integrations can vary between 1 and 30. If two tumour samples have an equally abundant dominant clone, comparing the clonality of integrations between these samples will be misleading, therefore for comparison between samples we normalize all integrations for a given sample such that the most-abundant integration is equal to 1 i.e., we annotate “normalized clonality values”.

To calculate the entropy (i.e., the measure of clonal outgrowth of each sample), the 50 highest clonality values *c*_1_, *c*_2_, …, *c*_50_ are transformed into probabilities *p*_*i*_

$$ {p}_i=\frac{c_i}{\sum \limits_{j=1}^{50}{c}_j} $$

and Shannon entropy *E* over a set of probabilities *p*_1_, *p*_2_, …, *p*_*n*_ is defined as:

$$ E=-\sum \limits_i{p}_i\log {p}_i $$

Lower values indicate a greater degree of clonal outgrowth.

## Supplementary information

**Additional file 1: Figure S1.** Step by step summary of LUMI-PCR library construction including all adapter and primer sequences. a) The figure depicts an LTR (blue bases) containing DNA fragment with a variable number of bases of genomic DNA (black X(X)nX bases) and a 5′ LTR sequence (blue). DNA is sheared by sonication. Overhanging ends are blunted, cleaned and then A-tailed (yellow highlighted bases) and cleaned. b) Adaptors containing unique indices (light green XXXXXXXXXX bases) and UMIs (dark green NNNNNNNN bases) are ligated to A-tailed DNA using T overhangs. c) Ligated fragments containing MuLV LTR sequence are bound by the LTR primary PCR primer. The first strand is synthesized from the LTR bound primer creating a lower strand that is compatible to the adapter primer. d) After the first strand of synthesis, the adapter primer and LTR primer can then amplify with exponential kinetics. Non-LTR containing fragments in the ligation are not amplified. e) A nested secondary PCR primer is used to add a second index sequence (light green XXXXXXXXXX bases) to the primary PCR product and further amplify the sequences. d) The secondary PCR products are quantitated, libraries are pooled and then loaded onto a MiSeq or HiSeq flow cell and clusters are generated. e) After cluster generation the first strand acts as the template for read 1 (the adapter end sequencing primer), index 1 (sequencing the LTR index added during the secondary PCR) and index 2 (the adapter index including the UMI sequenced from the flow cell primer). The index 2 read is a non-standard 18–20 bp, instead of the usual 10 bp, to include the UMI sequence. f) After strand regeneration, read 2 sequences the LTR-genome junction (using an LTR primer that can be placed at the junction or offset back from the junction). **Figure S2.** Graphical summary of informatics pipeline used to process reads into integration sites. Detailed step by step instructions for executing all scripts are available at https://github.com/anthonyuren/LUMI-PCR-pipeline/. **Figure S3.** Plate layouts for Beckman Biomek liquid handling workstation (below) Plate layouts for each program are listed on the next 4 pages. All programs begin with a box of tips loaded in the tip loader. Some programs require replacement of the tip box 30 min into the protocol. Detailed step by step protocols can be obtained by loading the .xpl files for each protocol into the Beckman Biomek software. **Table S1.** Diverse studies employ ligation-mediated PCR protocols for positioning of mobile genetic elements, viruses, transposons and transgene vectors. **Table S2.** Summary statistics for each library. Replicate libraries were prepared from a single spleen DNA sample from an MuLV-infected mouse. The number of reads, unique DNA fragments and integration sites are summarized.

**Additional file 2: Table S3.** The additional excel spreadsheet contains primer sequences, adapter sequences and example sample sheets.

**Additional file 3:** Protocol files for each step of the LUMI-PCR protocol using a Beckman Biomek workstation.

## Data Availability

All scripts and datasets are available from the github repository. http://www.github.com/anthonyuren/.

## Abbreviations

LTR: Long terminal repeat
NGS: next generation sequencing
MuLV: Murine leukemia virus
PCR: Polymerase chain reaction
UMI: Unique Molecular Identifiers
