# Supplementary material for: LUMI-PCR: an Illumina platform ligation-mediated PCR protocol for integration site cloning, provides molecular quantitation of integration sites
Source: Mob DNA. 2020 Feb 4;11:7. doi: 10.1186/s13100-020-0201-4 (PMC7001329; doi:10.1186/s13100-020-0201-4)
Supplement: Supplementary file 1 — Additional file 1: Figure S1. Step by step summary of LUMI-PCR library construction including all adapter and primer sequences. a) The figure depicts an LTR (blue bases) containing DNA fragment with a variable number of bases of genomic DNA (black X(X)nX bases) and a 5′ LTR sequence (blue). DNA is sheared by sonication. Overhanging ends are blunted, cleaned and then A-tailed (yellow highlighted bases) and cleaned. b) Adaptors containing unique indices (light green XXXXXXXXXX bases) and UMIs (dark green NNNNNNNN bases) are ligated to A-tailed DNA using T overhangs. c) Ligated fragments containing MuLV LTR sequence are bound by the LTR primary PCR primer. The first strand is synthesized from the LTR bound primer creating a lower strand that is compatible to the adapter primer. d) After the first strand of synthesis, the adapter primer and LTR primer can then amplify with exponential kinetics. Non-LTR containing fragments in the ligation are not amplified. e) A nested secondary PCR primer is used to add a second index sequence (light green XXXXXXXXXX bases) to the primary PCR product and further amplify the sequences. d) The secondary PCR products are quantitated, libraries are pooled and then loaded onto a MiSeq or HiSeq flow cell and clusters are generated. e) After cluster generation the first strand acts as the template for read 1 (the adapter end sequencing primer), index 1 (sequencing the LTR index added during the secondary PCR) and index 2 (the adapter index including the UMI sequenced from the flow cell primer). The index 2 read is a non-standard 18–20 bp, instead of the usual 10 bp, to include the UMI sequence. f) After strand regeneration, read 2 sequences the LTR-genome junction (using an LTR primer that can be placed at the junction or offset back from the junction). Figure S2. Graphical summary of informatics pipeline used to process reads into integration sites. Detailed step by step instructions for executing all scripts are available at https://github. [file 13100_2020_201_MOESM1_ESM.docx]

**Additional file 1: Figure S1** – Step by step summary of LUMI-PCR library construction including all adapter and primer sequences. a) The figure depicts an LTR (blue bases) containing DNA fragment with a variable number of bases of genomic DNA (black X(X)nX bases) and a 5’ LTR sequence (blue). DNA is sheared by sonication. Overhanging ends are blunted, cleaned and then A-tailed (yellow highlighted bases) and cleaned. b) Adaptors containing unique indices (light green XXXXXXXXXX bases) and UMIs (dark green NNNNNNNN bases) are ligated to A-tailed DNA using T overhangs. c) Ligated fragments containing MuLV LTR sequence are bound by the LTR primary PCR primer. The first strand is synthesized from the LTR bound primer creating a lower strand that is compatible to the adapter primer. d) After the first strand of synthesis, the adapter primer and LTR primer can then amplify with exponential kinetics. Non-LTR containing fragments in the ligation are not amplified. e) A nested secondary PCR primer is used to add a second index sequence (light green XXXXXXXXXX bases) to the primary PCR product and further amplify the sequences. d) The secondary PCR products are quantitated, libraries are pooled and then loaded onto a MiSeq or HiSeq flow cell and clusters are generated. e) After cluster generation the first strand acts as the template for read 1 (the adapter end sequencing primer), index 1 (sequencing the LTR index added during the secondary PCR) and index 2 (the adapter index including the UMI sequenced from the flow cell primer). The index 2 read is a non-standard 18-20 bp, instead of the usual 10 bp, to include the UMI sequence. f) After strand regeneration, read 2 sequences the LTR-genome junction (using an LTR primer that can be placed at the junction or offset back from the junction).

**Additional file 1: Figure S2 – Graphical summary of informatics pipeline used to process reads into integration sites.**

Detailed step by step instructions for executing all scripts are available at <https://github.com/anthonyuren/LUMI-PCR-pipeline/> .

**Additional file 1: Figure S3 – Plate layouts for Beckman Biomek liquid handling workstation (below)** Plate layouts for each program are listed on the next 4 pages. All programs begin with a box of tips loaded in the tip loader. Some programs require replacement of the tip box 30 minutes into the protocol. Detailed step by step protocols can be obtained by loading the .xpl files for each protocol into the Beckman Biomek software.

| **“Blunt Clean”** | | |
| --- | --- | --- |
| **P1** | **P4** | **P7** |
| Up-turned pipette box lid with ~30ml H_2_O (add after tip change) | Magnet plate | Empty Pyramid Plate (Cat#11510284) for tip wash |
| **P2** | **P5** | **P8** |
| Pyramid Plate (Cat#11510284) with 800μl 80% EtOH per well | Agencourt AMPure XP magnetic beads (Cat# A63882), **90μl**, in conical plate (Cat#30128575) stacked on a Nunc flat bottom 96 plate (Cat#269620) | Pyramid Plate (Cat#11510284) with 800μl H_2_O per well for tip wash |
| **P3** | **P6** | **P9** |
| DNA, **77.0μl**, in conical plate (Cat#30128575) stacked on a Nunc flat bottom 96 plate (Cat#269620) | Empty | DNA, **42.0μl**, Destination conical plate (Cat#30128575) stacked on a Nunc flat bottom 96 plate (Cat#269620) |

| **“A-tail Clean”** | | |
| --- | --- | --- |
| **P1** | **P4** | **P7** |
| Up-turned pipette box lid with ~30ml H_2_O (add after tip change) | Magnet plate | Empty Pyramid Plate (Cat#11510284) for tip wash |
| **P2** | **P5** | **P8** |
| Pyramid Plate (Cat#11510284) with 800μl 80% EtOH per well | Agencourt AMPure XP magnetic beads (Cat# A63882), **90.0μl**, in conical plate (Cat#30128575) stacked on a Nunc flat bottom 96 plate (Cat#269620) | Pyramid Plate (Cat#11510284) with 800μl H_2_O per well for tip wash |
| **P3** | **P6** | **P9** |
| DNA, **50.0μl**, in conical plate (Cat#30128575) stacked on a Nunc flat bottom 96 plate (Cat#269620) | Empty | DNA, **36.0μl**, Destination conical plate (Cat#30128575) stacked on a Nunc flat bottom 96 plate (Cat#269620) |

| **“Digest Clean”** | | |
| --- | --- | --- |
| **P1** | **P4** | **P7** |
| Up-turned pipette box lid with ~30 ml H_2_O (add after tip change) | Magnet plate | Empty Pyramid Plate (Cat#11510284) for tip wash |
| **P2** | **P5** | **P8** |
| Pyramid Plate (Cat#11510284) with 800 μl 80% EtOH per well | Agencourt AMPure XP magnetic beads (Cat# A63882), **60 μl**, in conical plate (Cat#30128575) stacked on a Nunc flat bottom 96 plate (Cat#269620) | Pyramid Plate (Cat#11510284) with 800 μl H_2_O per well for tip wash |
| **P3** | **P6** | **P9** |
| DNA, **60 μl**, in conical plate (Cat#30128575) stacked on a Nunc flat bottom 96 plate (Cat#269620) | Empty | DNA, **100 μl**, Destination conical plate (Cat#30128575) stacked on a Nunc flat bottom 96 plate (Cat#269620) |

| **“1^st^ Size Select”** | | |
| --- | --- | --- |
| **P1** | **P4** | **P7** |
| Empty | Magnet plate | Empty Pyramid Plate (Cat#11510284) for tip wash |
| **P2** | **P5** | **P8** |
| Empty | Agencourt AMPure XP magnetic beads (Cat# A63882), **60 μl**, in conical plate (Cat#30128575) stacked on a Nunc flat bottom 96 plate (Cat#269620) | Pyramid Plate (Cat#11510284) with 800 μl H_2_O per well for tip wash |
| **P3** | **P6** | **P9** |
| DNA, **100 μl**, in conical plate (Cat#30128575) stacked on a Nunc flat bottom 96 plate (Cat#269620) | Empty | DNA, **158 μl**, Destination conical plate (Cat#30128575) stacked on a Nunc flat bottom 96 plate (Cat#269620) |

| **“2^nd^ Size Select”** | | |
| --- | --- | --- |
| **P1** | **P4** | **P7** |
| Up-turned pipette box lid with ~30 ml H_2_O (add after tip change) | Magnet plate | Empty Pyramid Plate (Cat#11510284) for tip wash |
| **P2** | **P5** | **P8** |
| Pyramid Plate (Cat#11510284) with 800 μl 80% EtOH per well | Agencourt AMPure XP magnetic beads (Cat# A63882), **50 μl**, in conical plate (Cat#30128575) stacked on a Nunc flat bottom 96 plate (Cat#269620) | Pyramid Plate (Cat#11510284) with 800 μl H_2_O per well for tip wash |
| **P3** | **P6** | **P9** |
| DNA, **158 μl**, in conical plate (Cat#30128575) stacked on a Nunc flat bottom 96 plate (Cat#269620) | Empty | DNA, **30 μl**, Destination conical plate (Cat#30128575) stacked on a Nunc flat bottom 96 plate (Cat#269620) |

| **“1˚PCR Clean”** | | |
| --- | --- | --- |
| **Tip Station** |  |  |
| Biomek AP96 P250 Sterile Barrier Tips *3 (Cat#717253) |  |  |
| **P4** | **P4** | **P7** |
| Up-turned pipette box lid with ~30 ml H_2_O (add after tip change) | Magnet O | Empty Pyramid Plate (Cat#11510284) for tip wash |
| **P2** | **P5** | **P8** |
| Pyramid Plate (Cat#11510284) with 800 μl 80% EtOH per well | Agencourt AMPure XP magnetic beads (Cat# A63882), **50 μl**, in conical plate (Cat#30128575) stacked on a Nunc flat bottom 96 plate (Cat#269620) | Pyramid Plate (Cat#11510284) with 800 μl H_2_O for tip wash |
| **P3** | **P6** | **P9** |
| DNA, **50 μl**, in conical plate (Cat#30128575) stacked on a Nunc flat bottom 96 plate (Cat#269620) | Empty | DNA, **32 μl**, Destination conical plate (Cat#30128575) stacked on a Nunc flat bottom 96 plate (Cat#269620) |

| **“2˚PCR Clean”** | | |
| --- | --- | --- |
| **Tip Station** |  |  |
| Biomek AP96 P250 Sterile Barrier Tips *3 (Cat#717253) |  |  |
| **P4** | **P4** | **P7** |
| Up-turned pipette box lid with ~30 ml H_2_O (add after tip change) | Magnet O | Empty Pyramid Plate (Cat#11510284) for tip wash |
| **P2** | **P5** | **P8** |
| Pyramid Plate (Cat#11510284) with 800 μl 80% EtOH per well | Agencourt AMPure XP magnetic beads (Cat# A63882), **50 μl**, in conical plate (Cat#30128575) stacked on a Nunc flat bottom 96 plate (Cat#269620) | Pyramid Plate (Cat#11510284) with 800 μl H_2_O for tip wash |
| **P3** | **P6** | **P9** |
| DNA, **50 μl**, in conical plate (Cat#30128575) stacked on a Nunc flat bottom 96 plate (Cat#269620) | Empty | DNA, **100 μl**, Destination conical plate (Cat#30128575) stacked on a Nunc flat bottom 96 plate (Cat#269620) |

| **Mobile element/Vector** | **Host organism/cells/usage** | **Ref** |
| --- | --- | --- |
| LINE-1 retrotransposon | Identifying somatic retrotransposition in human lung cancer. | [doi](http://www.doi.org/10.1016/j.cell.2010.05.020) |
| LINE-1 retrotransposon | Identifying somatic retrotransposition in human colorectal cancer. | [doi](http://doi.org/10.1101/gr.201814.115) |
| Lentivirus | Tracing clonal evolution in xenografts of colon cancer using lentiviral integrants. | [doi](http://doi.org/10.1126/science.1227670) |
| Retrovirus vector | Gene trap in haploid human KBM7 cells to screen for essential genes. | [doi](http://doi.org/10.1126/science.aac7041) |
| ASLV/HIV/MuLV vectors | Monitoring expression based on integration sites placement relative to chromatin features. | [doi](http://dx.doi.org/10.3390/v10030116) |
| HTLV | Tracking integration frequencies for the study of clonal outgrowths in adult T-ALL. | [doi](http://doi.org/10.1186/gm568) |
| Retrovirus vector | Insertional mutagenesis in haploid hES cells to identify factors driving chemoresistance. | [doi](http://doi.org/10.18632/oncotarget.24305) |
| piggyBac | Screen for Gemcitabine resistance in pancreatic cancer cell line ASPC-1. | [doi](http://doi.org/10.1016/j.bbrc.2011.02.027) |
| piggyBac | Screen in developing mouse embryo brains for genes driving cortical malformation. | [doi](http://doi.org/10.1038/s41467-018-04880-8) |
| Various | Review article of somatic insertional mutagenesis screens in mouse models of cancer. | [doi](http://doi.org/10.1158/1541-7786.MCR-13-0244) |
| Sleeping Beauty | Germline mutagenesis in mice via ES cell route. | [doi](https://doi.org/10.1073/pnas.161071798) |
| Tol2 and maize Ac/Ds | Fluorescent protein fusion gene traps in zebrafish for visualization of proteins. | [doi](http://doi.org/10.1101/gad.174037.111) |
| P elements/piggyBac | Germline mutagenesis in *Drosophila*. | [doi](https://doi.org/10.1371/journal.pone.0010168) |
| LORE1 | Lotus Retrotransposon 1 endogenous integration survey and exon trap forward genetic screen. | [doi](http://doi.org/10.1111/j.1365-313X.2011.04826.x) |
| Barcoded *ura4*^+^ marker | Insertional mutagenesis screen of S.pombe. | [doi](http://doi.org/10.1186/1471-2164-13-161) |
| piggyBac | Insertional mutagenesis of *Plasmodium falciparum* to identify essential genes. | [doi](http://doi.org/10.1101/gr.200279.115) |
| Tn5 & mariner | Review article of insertional mutagenesis screens in bacteria. | [doi](http://doi.org/10.1007/s00253-015-7037-8) |

**Additional file 1: Table S1 – Diverse studies employ ligation-mediated PCR protocols for positioning of mobile genetic elements, viruses, transposons and transgene vectors.**

|  | library #918 | library #999 | library #1083 | library #1179 |
| --- | --- | --- | --- | --- |
| Reads | 216,430 | 282,732 | 292,974 | 424,902 |
| Q30 reads | 90,026 | 118,740 | 104,303 | 159,511 |
| Insert-genome junctions | 90,010 | 118,703 | 104,286 | 159,376 |
| Mapped reads pairs | 63,241 | 83,766 | 75,703 | 110,400 |
| Total UMIs | 4,911 | 10,085 | 8,954 | 12,859 |
| Total fragments | 1,197 | 2,811 | 2,509 | 3,172 |
| Total Inserts | 317 | 1,012 | 830 | 1,186 |

**Additional file 1: Table S2 - Summary statistics for each library.**

Replicate libraries were prepared from a single spleen DNA sample from an MuLV-infected mouse. The number of reads, unique DNA fragments and integration sites are summarized.
